# Seroprevalence of antibodies against Kaposi's sarcoma-associated herpesvirus among HIV-negative people in China

**DOI:** 10.1186/s13027-017-0142-9

**Published:** 2017-05-30

**Authors:** Tiejun Zhang, Zhenqiu Liu, Jun Wang, Veenu Minhas, Charles Wood, Gary M. Clifford, Na He, Silvia Franceschi

**Affiliations:** 1 Department of Epidemiology, School of Public Health, Fudan University, Shanghai, China; 2 Key Laboratory of Public Health Safety (Fudan University), Ministry of Education, Shanghai, China; 3 Nebraska Center of Virology and the School of Biological Sciences, University of Nebraska-Lincoln, Lincoln, USA; 4 International Agency for Research on Cancer, 69372 Lyon, Cedex 08 France

**Keywords:** Kaposi’s sarcoma associated herpesvirus, Prevalence, China

## Abstract

**Background:**

Little information on the prevalence of Kaposi’s sarcoma associated herpesvirus (KSHV) among HIV-negative individuals is available from Asia.

**Methods:**

In the present study, we report findings from a new survey of KSHV in 983 HIV-negative male migrants from Shanghai and their combination with previous similar surveys of 600 female migrants, 600 female sex-workers (FSW), 1336 sexually transmitted infection (STI) clinic male patients, 439 intravenous drug-users (IVDU), and 226 men having sex with men (MSM) from China. KSHV-specific antibodies against latent and lytic antigens were assessed using Sf9 and BC3 monoclonal immunofluorescence assay. Age-adjusted prevalence ratios (PR) and 95% confidence interval (95% CI) for KSHV-positivity were estimated using Poisson regression.

**Results:**

In total, 4184 HIV-negative participants were included. KSHV prevalence ranged from 9.8% (95% CI: 7.9%-11.7%) in male migrants to 32.3% (95% CI: 24.1%-34.1%) in MSM. IVDU show intermediate level (17.5%, 95%CI: 14.1%-21.4%). KSHV was associated with syphilis (PR = 2.03, 95% CI: 1.38-2.98) in MSM but not in other groups. An association with human herpes virus 2 was also found among MSM (PR = 1. 83, 95%: 1.22-2.75) but not in migrant workers or FSW.

**Conclusions:**

KSHV prevalence in HIV-negative heterosexuals, FSW, and STI male patients from China is approximately 10%, but 2- and 3-fold higher in IVDU and MSM, respectively. Associations of KSHV with STIs among MSM only suggest that sexual transmission of the virus is important in MSM but not in heterosexuals.

## Background

Kaposi’s sarcoma-associated herpesvirus (KSHV), also referred to as human herpesvirus-8 (HHV-8), is the etiologic agent of KS, primary effusion lymphomas and multicentric Castleman’s disease (IARC 2012 Vol 100B). Contrary to other herpesviruses, prevalence of KSHV varies enormously among regions and sub-populations. It is generally low (<10%) in the general population in Northern Europe, the United States and Asia, [1], moderate in the Mediterranean region (10-30%), and high in sub-Saharan Africa (>30%) (IARC 2012 Vol 100B).

By far the strongest risk factor for KS is immunosuppression (IARC 2012 Vol 100B). KS risk steeply increases with the decline in CD4+ count and diminishes rapidly after the starting of combined antiretroviral therapy (cART) [2]. A meta-analysis showed that HIV infection was also associated with a two-fold increase in KSHV prevalence, and a four-fold increase among HIV-positive men having sex with men (MSM) [3]. In respect to regional variations, KSHV prevalence in HIV-infected people increases by two-times or more in Western countries and by 56% in sub-Saharan Africa but data on the association of the two infections from Asia are scant [3, 4].

Like other herpesviruses KSHV is primarily transmitted via saliva and acquisition in KS-endemic areas typically occurs in childhood from the mother or horizontally. Blood-borne transmission exists among injection drug users (IVDU) and blood recipients but it is rare. [5, 6] Sexual transmission is considered important in MSM but has not been demonstrated in heterosexual individuals. [7–11].

To throw more light on the prevalence and the modalities of KSHV transmission among HIV-negative individuals in China, we carried out a new survey of KSHV prevalence in male migrant workers. We also combined the new survey with the findings from five additional HIV-negative Chinese subpopulations that we had previously studied using the same study protocol [12–15].

## Methods

### Male migrant workers

From May to October 2015 we conducted a survey on the characteristics and prevalence of various infections among male migrant workers in Shanghai, China (hereinafter referred to as male migrants). The included migrants were men aged 18 years or older, had lived in the community for more than 3 months and were able to provide a written informed consent. One thousand male migrants were invited and 983 of them fully complied with study requirements (median age = 27 years; range: 18-66). They were interviewed in private by trained staff in Mandarin language using an anonymized questionnaire that included information on socio-demographic characteristics and sexual behaviours. Venous blood was collected using sterilized needles and tubes, and transferred to laboratory within 2 h after collection while maintaining a cold chain. Plasma samples were stored at −80 °C until serological testing.

### Serology testing

#### KSHV testing

Plasma samples were tested by two monoclonal immunofluorescence assays (mIFAs) that target KSHV latent and lytic antigens [16]. Briefly, two serology tests were performed: first, BC-3 cells (KSHV positive and Epstein-Barr virus negative B cell line, American Type Culture Collection, Manassas, VA), stimulated by tetradecanoyl phorbol acetate (TPA), were fixed and permeabilized and used for an enhanced mIFA. Second, *Spodoptera frugiperda* clone 9 expressing 3 KSHV proteins, ORF73, ORF65 and ORF-K8.1, was used. Sera taken from two KS patients and a normal person were as positive and negative controls, respectively, in each assay. The two assays were then compared, and only samples which were positive for both BC-3 and Sf9 assay at a standard serum dilution of 1:40 were considered KSHV-seropositive. The testing protocol used in our present study have been validated and shown to have sensitivity of 93.9% and specificity of 96.3% [16]. To guarantee the quality, all slides were monitored for every batch and were read independently by two experienced laboratory workers.

#### Syphilis testing

A rapid plasma reagent test (Span Diagnostics Ltd., Surat, India), was used and confirmed by the *Treponema pallidum hemagglutination test* (TPHA, Syphagen TPHA, Biokit, Spain).

#### HSV-2 testing

HSV-2 IgG antibodies were tested using an ELISA assay (HerpeSelect 2 ELISA IgG Kit, Focus Technologies, CA, USA). Equivocal samples were retested using another ELISA kit (HerpeSelect 2 ELISA IgG Kit, Euroimmun, Lübeck, Germany).

#### HCV testing

Anti-HCV immunoglobulin G (IgG) antibodies were tested by third-generation ELISA (Wantai Biomedical, Beijing, China).

#### HIV testing

HIV antibodies were assessed using ELISA (Vironostika HIV Uni-Form II plus O ELISA Kit, Biomerieux, Netherland) and confirmed by a western blot assay (Genelabs Diagnostic, Singapore).

### Pooled analysis

The data from the male migrants study were pooled with those from five additional subpopulations that we had studied between 2010 and 2015 using similar questionnaires and the same serological assays described above. Briefly, we included 600 female migrant workers (age:29.34 ± 8.44), referred to as female migrants [15]; 600 female sex workers (FSW) (age: 26.47 ± 6.84) [15]; 1336 men attending sexually transmitted infection clinics, referred to as STI men (age: 37.39 ± 14.13) [12]; 439 intravenous drug users (IVDU) (age:45.39 ± 88.41) [14]; and 226 men having sex with men (MSM) (age: 27.79 ± 7.01) [13]. Thus, a total of 3201 HIV negative individuals in addition to the aforementioned 983 HIV negative male migrants were finally included.

All studies had been approved by the Institutional Review Board (IRB) of Fudan University, Shanghai. Informed consent was obtained from all subjects, all study protocols and procedures were in accordance with the Declaration of Helsinki.

### Statistical analysis

The prevalence of KSHV, syphilis, and when available, HSV2, was examined separately in the six available subpopulations and eventually combined into three groups: 1) heterosexuals, i.e., migrants of both sexes, FSW, and STI men; 2) IVDU; and 3) MSM (including 27 MSM who were formerly included in the STI survey [12]). On account of the limitations and inconsistencies of sexual history in study questionnaires, the prevalence of syphilis and HSV2 was preferred to number of sexual partners as proxies of sexual activity. Ninety-five percent confidence intervals (95% CI) of prevalence were computed according to the normal approximation to the binomial distribution. Prevalence ratios (PR) and 95% CI for KSHV positivity were estimated by the Poisson regression models with robust variance. PRs were adjusted by age group or by age group and subpopulation as indicated. Risk trends were assessed by considering categories as continuous variables. All statistical analyses were carried out using the SAS System for Windows (Cary, NC, USA), version 8.0.

## Results

**Fig. 1:**
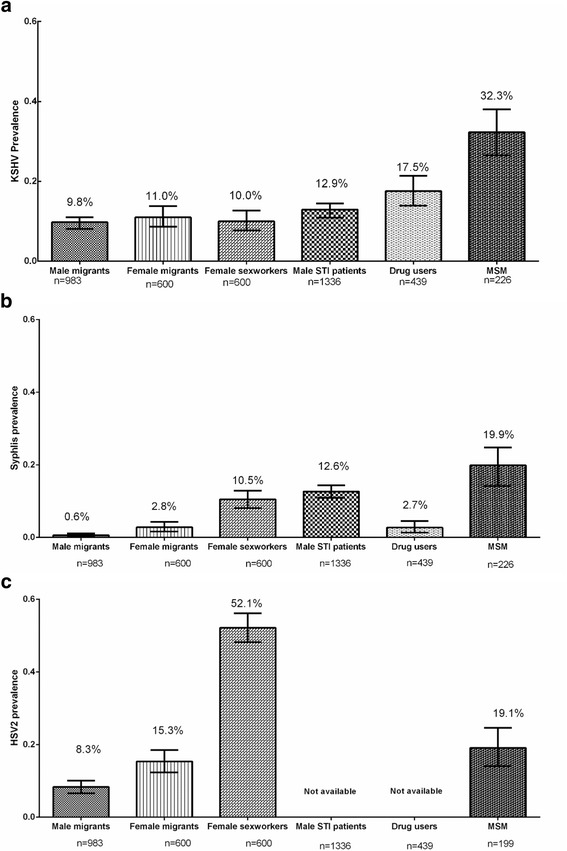
Prevalence of KSHV (**a**), Syphilis (**b**) and HSV2 (**c**) among different HIV-negative subpopulations China, 2010-2015

Among male migrants, only older age (PR for ≥35 vs. <25 = 0.58, 95% CI: 0.34-1.01) and higher education (PR versus primary education = 0.43, 95% CI: 0.23-0.82, for middle school; and 0.29, 95% CI 0.11-0.80, for college attendance) were associated with KSHV-positivity (Table 1). Only 35% of male migrants were living with a spouse or female partner. The prevalence of antibodies among male migrants was 9.8% for KSHV (95% CI: 7.9%-11.7%), 8.3% for HSV2 (95% CI: 6.7%-10.2%), 0.6% for syphilis (95% CI: 0.2%-1.3%) (Fig. 1) and 0.4% for HCV (95% CI: 0.1%-0.9%) (data not shown).

**Table 1:** Sociodemographic characteristics and KSHV infection among 983 male migrants, Shanghai, 2015

Characteristic	No. (%)	KSHV+ (%)	PR (95%CI)^a^
Age (years)			
18-24	354 (36.0)	39 (11.0)	1.00
25-34	363 (36.9)	40 (11.0)	1.00 (0.66-1.52)
≥ 35	266 (27.1)	17 (6.4)	0.58 (0.34-1.01)
*x* ^2^_*trend*_ = 3.328, *P* = 0.068			
School education			
Primary or lower	53 (5.4)	9 (17.0)	1.00
Middle school	846 (86.1)	81 (9.6)	0.43 (0.23-0.82)
College	84 (8.5)	6 (7.1)	0.29 (0.11-0.80)
*x* ^2^_*trend*_ = 3.028, *P* = 0.081			
Marital status			
Never married	463 (47.1)	52 (11.2)	1.00
Ever married	520 (52.9)	44 (8.5)	0.83 (0.51-1.38)
Stay in Shanghai (yrs)			
< 1	235 (23.9)	23 (9.8)	1.00
1-2	278 (28.3)	32 (11.5)	1.17 (0.71-1.93)
≥ 3	470 (47.8)	41 (8.7)	1.01 (0.59-1.69)
*x* ^2^_*trend*_ = 0.386, *P* = 0.534			
Monthly income (yuan RMB)			
≤ 2000	43 (4.4)	7 (16.3)	1.00
2001-4000	677 (68.9)	59 (8.7)	0.54 (0.26-1.01)
> 4000	263 (26.8)	30 (11.4)	0.72 (0.33-1.57)
*x* ^2^_*trend*_ = 0.101, *P* = 0.750			
Living with			
Alone	163 (16.6)	14 (8.6)	1.00
With a spouse or partner	348 (35.4)	36 (10.3)	1.31 (0.73-2.32)
With others	472 (48.0)	46 (9.7)	1.06 (0.58-1.91)

^a^Adjusted by age group

Figure 1 shows variations in the prevalence of KSHV, syphilis and HSV2 in male migrants and in the other five sub-populations. KSHV prevalence in male and female migrants, FSW, and STI men was approximately 10% and significantly lower than in MSM (32.3%; 95% CI: 24.1%-34.1%). IVDU showed an intermediate KSHV prevalence (17.5%; 95% CI: 14.1%-21.4%). The prevalence of syphilis ranged from 0.6% (95% CI: 0.2%-1.3%) in male migrants to 19.9% (95% CI: 14.9%-25.7%) in MSM and was also significantly elevated (>10%) in FSW and STI men (Fig. 1b). HSV2 prevalence was significantly higher among FSW (52.1%; 95% CI: 48.1%-56.2%) and MSM (19.1%; 95% CI: 13.9%-25.3%) as compared with male (8.3%) and female migrants (15.3%) but it was not available for STI men and IVDU.

The relationship between KSHV prevalence and syphilis and HSV2 infection in each subpopulation and pooled PRs according to sexual orientation (heterosexual or MSM) or IVDU status is shown in Table 2. Syphilis was significantly associated with KSHV-positivity among MSM (PR = 2.03, 95% CI: 1.38-2.98) but not in any group of heterosexual men or women. The pooled PR in heterosexual subpopulations was 0.97; (95% CI: 0.67-1.38). Similar results were found for HSV2, i.e., PR = 1.83 (95%:1.22-2.75) in MSM but 1.22 (95% CI: 0.89-1.67) in the combination of migrant workers and FSW.

**Table 2:** Prevalence ratios (PR) and 95% confidence intervals (95% CI) of KSHV by prevalence of syphilis and HSV2 in individual subpopulations and pooled PR among heterosexuals, China 2010-2015

	Syphilis			HSV2^a^		
	No.	KSHV+ (%)	PR (95% CI)^b^	No.	KSHV+ (%)	PR (95% CI)^b^
Male migrants						
Negative	977	95 (9.7)	1.00	901	87 (9.7)	1.00
Positive	6	1 (16.7)	1.52 (0.25-9.41)	82	9 (11.0)	1.13 (0.59-2.16)
Female migrants						
Negative	583	65 (11.1)	1.00	508	55 (10.8)	1.00
Positive	17	1 (5.9)	0.54 (0.08-3.65)	92	11 (12.0)	1.12 (0.61-2.06)
Female sex workers						
Negative	537	56 (10.4)	1.00	287	25 (8.7)	1.00
Positive	63	4 (6.3)	0.63 (0.28-1.75)	313	35 (11.2)	1.31 (0.81-2.12)
Male STI patients						
Negative	1167	147 (12.6)	1.00	--	--	--
Positive	169	25 (14.8)	1.07 (0.71-1.62)	--	--	--
Total heterosexuals^c,d^						
Negative	3264	363 (11.1)	1.00	1696	167 (9.8)	1.00
Positive	255	29 (11.4)	0.97 (0.67-1.38)	487	55 (11.3)	1.22 (0.89-1.67)
Intravenous drug users						
Negative	427	76 (17.8)	1.00	--	--	--
Positive	12	1 (8.3)	0.51 (0.08-3.40)	--	--	--
Men who have sex with men^e^						
Negative	181	48 (26.5)	1.00	161	46 (28.6)	1.00
Positive	45	25 (55.6)	2.03 (1.38-2.98)	38	20 (52.6)	1.83 (1.22-2.75)

^a^HSV2 findings are based on 2183 individuals only as the test was not done in 1336 STI patients

^b^Adjusted by age

^c^Additionally adjusted by group

^d^Total heterosexuals refer to the Male migrants, Female migrants, Female sex workers and Male STI patients, not Intravenous drug users or Men who have sex with men

^e^HSV2 status was unknown in 27 MSM

## Discussion

Our study of HIV-negative men and women from China shows that KSHV prevalence is approximately 10% in migrant workers of both sexes, FSW, and STI men but 2- and 3-fold higher in IVDU and MSM, respectively. We also found a substantial variability in the prevalence of syphilis (with peaks of >10% in FSW, STI men, and MSM) and of HSV2 infection which was detected in more than half of FSW. However, an association of KSHV with the two STIs was only present among MSM.

Epidemiological data regarding KSHV prevalence and KS incidence in HIV-negative populations in Asia is sparse. Compare to the relatively high prevalence of KSHV, age-standardized incidence rates of KS in the fourteen highest-quality cancer registries from China were consistently below 0.3 per 100,000 in both sexes and no KS cases was recorded in many registries in the 2003-2007 period [17]. Likewise, similarly low KS incidence is found in other cancer registries from Asia, except for a few registries in Turkey, Israel, and Qatar [17]. Under-report of KS in low/medium-income countries is a possibility but the likeliest explanation of the coexistence of moderate KSHV prevalence with very low incidence of the disease in China is the relative rarity of HIV infection in the general population. The rapid expansion of population-based cancer registries in China [18] will allow us to understand whether any little studied Chinese region shows a higher KS burden. Indeed, a large meta-analysis suggested the existence of areas, notably in the western part of China, in which KSHV prevalence is 20% or more [4].

Unfortunately, the comparisons of KSHV prevalence from different studies is hampered by the substantial variations in the sensitivity of KSHV assays [19], a problem that does not exist in our present surveys in which two mIFAs were jointly used in one centralized high-quality laboratory [16]. This strength allowed us to explore more reliably the possibility of sexual transmission of KSHV in HIV-negative individuals. Firstly, we found no significant variations in KSHV prevalence among subpopulations of heterosexuals with low-risk (migrant workers of both sexes) or high-risk (FSW and STI men) sexual behavior. Secondly, we could demonstrate that two STIs (syphilis and HSV2) were exclusively predicting the prevalence of KSHV in MSM.

MSM are at strongly increased risk of KSHV in all studied populations regardless of HIV status [3, 13, 20]. Number of sexual partners, anal and/or oro-genital intercourse, and history of STIs are recognized risk factors for the acquisition of the infection in MSM [7, 9, 21].

KSHV prevalence in our study was also moderately increased but not associated with the prevalence of syphilis in IVDU. Other studies have provided evidence that KSHV can be transmitted by blood or blood products [22–24]. The longer the duration of injection drug use the higher was the risk of KSHV infection independently from sexual behavior or demographic differences.

We had already reported lack of association of KSHV prevalence with the number of sexual partners female migrants and FSW [15] and in STI men [12] but each individual survey was relatively small and self-reported number of sexual partners is not necessarily accurate in China. The current pooled analysis is based on two objective measures of sexual habits (syphilis and HSV2) and represents therefore much stronger evidence of the low sexual transmissibility of KSHV in different heterosexual subpopulations than self-reported sexual habits.

## Conclusions

In conclusion, the present study revealed that KSHV in China is moderately prevalent among HIV negative heterosexual individuals, but highly prevalent among MSM. These results may provide insights into potential future public health impact of KSHV in the HIV negative population. The results from current study highlight opportunities and challenges in this field of study. Definitely further studies are needed, in order to clarify the potential risk of KSHV transmission in Chinese population.

## Abbreviation

CI: Confidence interval
FSW: Female sex-workers
HCV: Hepatitis C virus
HIV: Human immunodeficiency virus
HSV2: Herpes simplex virus type 2
IVDU: Intravenous drug-users
KSHV: Kaposi’s sarcoma associated herpesvirus
MSM: Men having sex with men
PR: Prevalence ratios
